# Isoform-specific expression of the Coxsackie and adenovirus receptor (CAR) in neuromuscular junction and cardiac intercalated discs

**DOI:** 10.1186/1471-2121-5-42

**Published:** 2004-11-08

**Authors:** Christian A Shaw, Paul C Holland, Michael Sinnreich, Carol Allen, Kerstin Sollerbrant, George Karpati, Josephine Nalbantoglu

**Affiliations:** 1 Department of Neurology & Neurosurgery, McGill University and Montreal Neurological Institute, Montreal, Canada; 2 Ludwig Institute for Cancer Research (Stockholm Branch), Karolinska Institute, Stockholm, Sweden

## Abstract

**Background:**

The Coxsackie and adenovirus receptor (CAR) has a restricted expression pattern in the adult. In skeletal muscle, although CAR is expressed in immature fibers, its transcript levels are barely detectable in mature muscle. This is in contrast to the robust expression observed in the heart. However, both heart and skeletal muscle are susceptible to infection with the Coxsackie B virus which utilizes primarily CAR for cellular internalization. The specific point of viral entry in skeletal and heart muscle remains unknown.

**Results:**

Using antibodies directed against the extracellular and the cytoplasmic domains of CAR, we show CAR in normal human and mouse skeletal muscle to be a novel component of the neuromuscular junction. In cardiac muscle, CAR immunoreactivity is observed at the level of intercalated discs. We demonstrate a single isoform of CAR to be expressed exclusively at the human neuromuscular junction whereas both predominant CAR isoforms are expressed at the intercalated discs of non-diseased human heart.

**Conclusion:**

The localization of CAR to these important junctional complexes suggests that CAR may play both a structural and a regulatory role in skeletal and cardiac muscle, and that these complexes may serve as a point of entry for Coxsackie B virus.

## Background

The Coxsackie and adenovirus receptor (CAR) [1,2], a transmembrane protein of the immunoglobulin super-family, serves as a receptor for adenovirus (Ad) subgroups A, C, D, E and F [3] as well as Coxsackie B viruses (CVB) [4]. CAR is a highly conserved protein with two predominant isoforms, produced through differential splicing, and having cytoplasmic domains of either 107 residues (ending in SIV) or 94 residues (ending in TVV) [2,5]. The extracellular domain mediates homophilic cell adhesion [6-8] and ectopically-expressed CAR localizes to homotypic intercellular contacts [8]. The expression of CAR is regulated developmentally [6,9-12] as well as in a tissue-specific manner [2,5]. To date, most studies on CAR expression in the adult have resorted to analysis of transcript levels. These have revealed that the pattern of tissue-specific expression differs between humans and mice. In humans, a predominant transcript of ~6 – 6.5 kb is observed in heart, testis, prostate and pancreas while much less expression is detected in liver, brain, colon and small intestine. In the mouse on the other hand, the most abundant expression is in liver, kidney, lung and heart.

**Figure 1:**
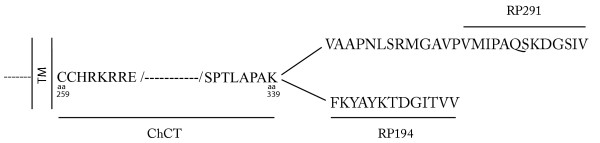
**Depiction of the CAR sequences recognized by the anti-C-terminal antibodies. **The chicken antibody ChCT was raised against a fusion protein containing C-terminal sequence common to both CAR isoforms (from aa 259 – 339). The rabbit antibodies (RP291 and RP194) were raised against peptides that were specific to each isoform. The diagram does not show the immunogen of the rabbit antibody 2240 which is the extracellular N-terminal domain of murine CAR. Note that each antibody has a distinct recognition sequence. TM – transmembrane sequence.

Interest in CAR stems from its function as the primary high affinity receptor for Ad serotype 5, the most commonly used adenoviral vector in gene therapy protocols. CAR expression is the main determinant in gene transfer to normal tissue as ectopic expression of CAR in transgenic mice leads to several magnitudes of increase in adenovirus transducibility of tissues that are otherwise refractory to Ad-mediated gene expression [13-17]. As well, although decay accelerating factor (DAF, CD55) was the first described CVB receptor [18,19], CAR is necessary and sufficient for CVB infection *in vitro *[20]. Thus, the expression levels of CAR may also govern the susceptibility to CVB diseases and the pathological consequences of CVB viral infection. In this context, acute viral myocarditis and myositis are inflammatory diseases affecting cardiac and skeletal muscle that can result from infection by the Coxsackie B virus. In both humans and rodents, heart is among the tissues showing the greatest abundance of CAR transcript while its transcripts are barely detectable in skeletal muscle even with the more sensitive reverse-transcriptase (RT)-PCR-based assay [21]. In contrast to heart, DAF expression is absent in mature skeletal muscle [22]. Despite the absence of DAF and low CAR transcript levels, skeletal muscle is nevertheless susceptible to Coxsackie virus-induced myositis. Indeed, human patients suffering from inflammatory muscle diseases have tested positive for CBV RNA [23]. This suggests that the low CAR transcript level in skeletal muscle may produce functional receptor. Therefore, to examine CAR localization in skeletal and cardiac muscle, we used antibodies directed against the extracellular domain of CAR [21] as well as antibodies that can differentiate between the two major CAR isoforms [24] with alternate 3' splicing [ending in the amino acids SIV or TVV] [2,5] [Fig. 1].

## Results and Discussion

**Figure 2:**
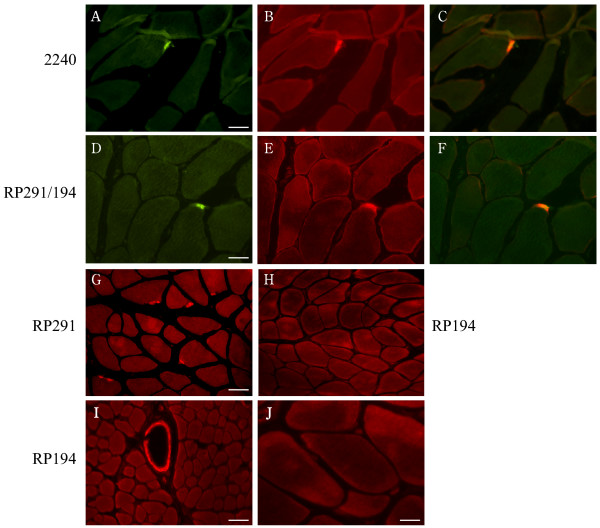
**Immunolocalization of CAR to the neuromuscular junction in human skeletal muscle**. Frozen sections of human muscle were incubated with Alexa Fluor-conjugated α-bungarotoxin and antibodies to CAR as described in Materials and Methods. Panels **A **and **D **show α-bungarotoxin staining of acetylcholine receptors at neuromuscular junctions (green). Panel **B **shows immunofluorescent staining of the section in panel **A **with a polyclonal antibody (ab 2240) to the extracellular domain of CAR (red). Panel **E **shows immunofluorescent staining of the section in panel **D **with a mixture of the isoform-specific C-terminal antibodies RP194 and RP 291 (red). Panel **C **is a merge of panels **A **and **B**. Panel **F **is a merge of panels **D **and **E**. These merges [**C, F**] show that CAR colocalizes with α-bungarotoxin at neuromuscular junctions (in yellow). Of the two C-terminal antibodies, only RP291 (**G**) demonstrates the typical neuromuscular junction staining while signal is absent when sections are incubated under similar conditions with RP194 (**H**) although RP194 does label blood vessels (**I**). Sections incubated with secondary antibody alone did not reveal any signals (**J**). Bar = 25 μm (A, D); 50 μm (G, I).

**Figure 3:**
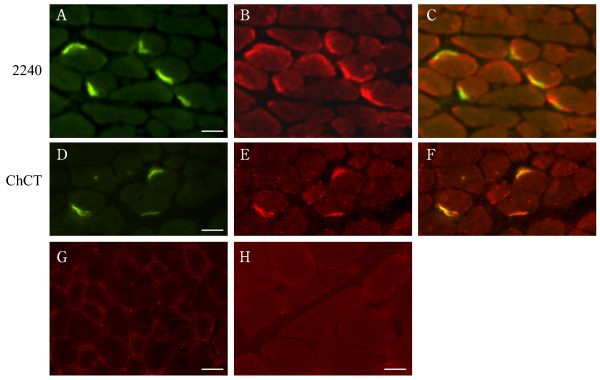
**Immunolocalization of CAR to the neuromuscular junction in mouse skeletal muscle**. Frozen sections of mouse muscle were incubated with Alexa Fluor-conjugated α-bungarotoxin and antibodies to CAR as described in Materials and Methods. Panels **A **and **D **show α-bungarotoxin staining of acetylcholine receptors at neuromuscular junctions (green). Panel **B **shows immunofluorescent staining of the section in panel **A **with a polyclonal antibody (ab 2240) to the extracellular domain of CAR (red). Panel **E **shows immunofluorescent staining of the section in panel **D **with a chicken polyclonal antibody (ChCT) directed against a C-terminal portion of CAR conserved in both CAR isoforms (red). Panel **C **is a merge of panels **A **and **B**. Panel **F **is a merge of panels **D **and **E**. These merges [**C, F**] show that CAR colocalizes with α-bungarotoxin at murine neuromuscular junctions (in yellow). Sections incubated with the secondary antibodies alone did not give any signal – anti-rabbit IgG [**G**], anti-chicken IgY [**H**]. Bar = 35 μm

To immunolocalize CAR, frozen sections of normal human muscle biopsies were probed with polyclonal antibodies raised against the extracellular domain of CAR [21] [ab 2240], or against the cytoplasmic tail, antibodies which specifically recognize the two predominant isoforms of human CAR (referred to as SIV [ab RP291] and TVV [ab RP194] respectively) [24]. We localized CAR exclusively to the neuromuscular junction in human skeletal muscle (Fig. 2) where CAR immunoreactivity coincided with acetylcholine receptors as revealed by α-bungarotoxin binding. Similarly, in mature murine skeletal muscle, CAR expression was restricted to the neuromuscular junction (Fig. 3). Interestingly, we find CAR expression at the neuromuscular junction to be isoform specific, with the CAR SIV isoform accounting for all CAR immunoreactivity (Fig. 2G). In contrast, the TVV isoform is present in the vasculature (Fig. 2I). CAR is therefore identified as a novel component of the adult neuromuscular junction, joining other homotypic cell adhesion molecules such as N-cadherin and neural cell adhesion molecule (N-CAM) which have been localized previously to this specialized site. As with NCAM [25], although CAR is uniformly expressed in immature mouse muscle (data not shown), its expression becomes downregulated within a few weeks of birth [21] and its localization is confined to the neuromuscular junction. As well, in skeletal muscle undergoing regeneration, as is the case in the dystrophic *mdx *mouse, CAR is re-expressed [21] as is NCAM [26].

**Figure 4:**
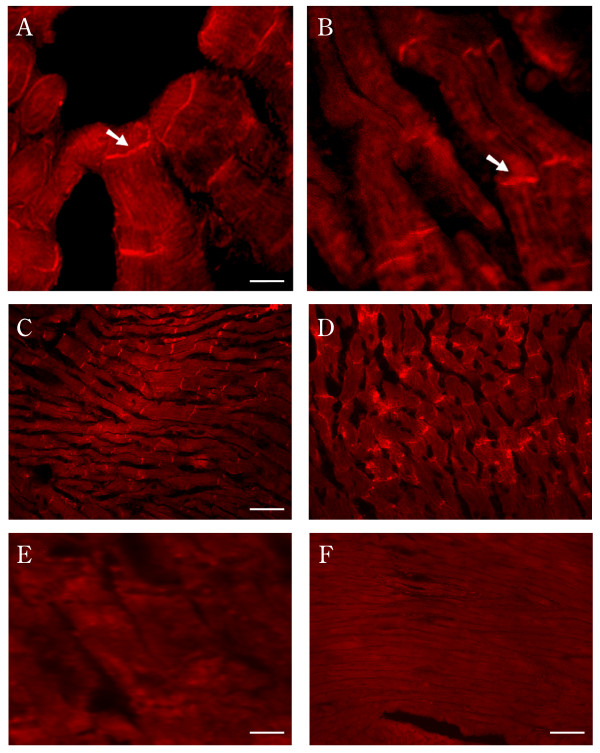
**Immunolocalization of CAR to intercalated discs in human and murine cardiac muscle. **Human [**A, B**] and murine [**C, D**] cardiac muscle sections were reacted with the polyclonal anti-C-terminal antibodies RP194 [**A, C**] and RP291 [**B, D**]. Note the intense staining of both isoforms at the human intercalated discs (arrow) [**A, B**] and murine intercalated discs [**C, D**]. Neither the human cardiac tissue [**E**] nor the murine cardiac muscle [**F**] gave any signal when incubated with the anti-rabbit IgG. Bar = 10 μm (A, E); 30 μm (C, F).

**Figure 5:**
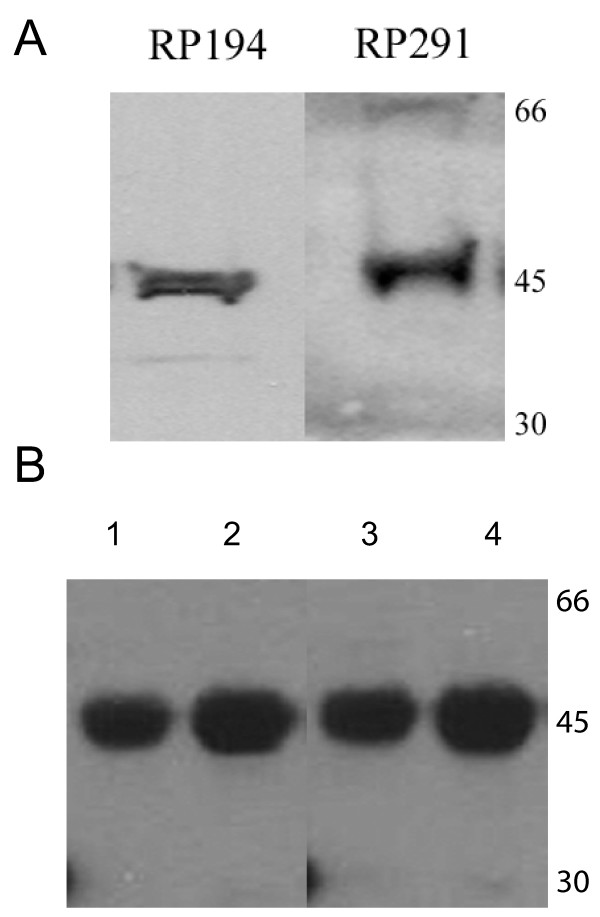
**Murine cardiac muscle homogenates express both isoforms of CAR. A. **Immunoblot analysis of cardiac muscle homogenates (10 μg) reveals that a single polypeptide of 46 kDa is detected by the anti-C-terminal antibodies recognizing the two different CAR isoforms (RP194 and RP291). **B. **Murine cardiac homogenates were immunoprecipitated with either RP194 (lanes 1,2) or RP291 (lanes 3,4) followed by immunoblotting with the chicken ChCT antibody that recognizes the C-terminal portion of CAR that is common to both isoforms. The ChCT antibody detected as a single band the increasing amounts of CAR that was immunoprecipitated with increasing amounts of the rabbit polyclonal antibodies (2 μl, lanes 1,3; 4 μl, lanes 2,4).

CAR has been suggested to serve as a structural component at the intercalated discs in murine cardiac muscle [8] and recently confirmed to reside primarily at the intercalated discs in both rat heart and neonatal cultured cardiomyocytes of the rodent [12]. Using antibodies directed to the specific SIV and TVV isoforms of the receptor as described above, we localized CAR to the intercalated discs (Fig. 4), demonstrating for the first time expression of both receptor isoforms in human and murine heart. To confirm the presence of both isoforms, murine cardiac muscle homogenates were analyzed by SDS-PAGE followed by electrotransfer to nitrocellulose membranes. This immunoblot analysis revealed the presence of both the SIV and TVV isoforms (Fig. 5A). Furthermore, when murine cardiac muscle homogenates were immunoprecipitated with either RP194 or RP 291 rabbit polyclonal antibodies, Western blot analysis of the resultant fractions with a chicken antibody against CAR C-terminal domain showed a single 46 kDa band (Fig. 5B), confirming the specificity of the antibodies for CAR.

Both of the cytoplasmic variants contain a PDZ recognition motif at their distal end, implicating CAR as a putative member of multiprotein complexes. This is indeed the case in polarized epithelial cells in which CAR is expressed at the tight junction where it associates with the tight junction scaffolding protein ZO-1 [8] and contributes to maintenance of transepithelial resistance. The localization of CAR to cardiac intercalated discs is in agreement with CAR having a structural or regulatory role as a transmembrane member of junctional complexes. The intercalated discs are composed of at least three structurally distinct cellular junctions – desmosomes, the adherens junctions, and gap junctions [27]. The SIV and TVV isoforms may localize to separate components of the intercalated disc, or given that CAR has been implicated as a transmembrane component in tight junctions [8], both isoforms may be localized to the adherens junction, a structure analogous to tight junctions.

It is interesting that a correlation can be drawn between disease incidence and expression levels of the CAR receptor. Cases of viral myocarditis in the human population outnumber those of myositis. This may be attributable to the difference of endogenous CAR expression between the two tissues. Considering that induction of CAR accompanies myocarditis [28] and its dramatic upregulation has recently been demonstrated in patients suffering from multiple diseases of the heart [29] including dilated cardiomyopathy, a pathological phenotype linked to persistent acute myocarditis, upregulation of the receptor may render the heart even more susceptible to further viral infection. Conversely, the low level of endogenous CAR expression in skeletal muscle may safeguard against the wide-spread viral infection seen in myocarditis and may be responsible for the less severe clinical features of myositis.

## Conclusions

CAR is a novel member of the neuromuscular junction. In cardiac muscle, both CAR isoforms are found at the intercalated discs. The localization of CAR to these important junctional complexes suggests that CAR may play both a structural and a regulatory role in skeletal and cardiac muscle, and that these complexes may serve as a point of entry for Coxsackie B virus.

## Methods

### Antibodies

Figure 1 depicts the sequences used as immunogen to generate the various antibodies. There was no overlap (i.e. common epitopes) between any of the antibodies. The rabbit polyclonal antibodies used in this study were described previously [21,24]. Briefly, the N-terminal polyclonal antibody (ab 2240) was prepared against a His-tagged fusion protein which encoded amino acid residues 22–208 of the extracellular domain of mouse CAR [21]. This antiserum cross-reacts with human CAR on Western blots and in indirect immunofluorescence. The two C-terminal polyclonal antibodies [24] were generated using peptides encompassing the last 13 amino acids of the two predominant human CAR isoforms [2,5]. Antiserum RP194 was raised against the sequence FKYAYKTDGITVV while RP291 was raised against the sequence VMIPAQSKDGSIV. Both these antisera cross-react with the mouse CAR homologs (the peptides are conserved 100% between the two species [30]). All antisera were affinity purified prior to use.

To raise the chicken anti-CAR antibody (ChCT), purified His-tagged fusion protein encoding the C-terminal portion of CAR that is common to both isoforms (amino acids 259–339) was emulsified in an equal volume of TiterMax Gold adjuvant (CytRx Corp., Norcross, GA) and injected intramuscularly into chickens. One month post-injection, IgY antibodies to CAR were obtained from the eggs of injected chickens and subjected to affinity purification.

### Immunocytochemistry

Immunolabelling was performed using standard techniques. Briefly, frozen sections (5 μm) of normal human skeletal and cardiac muscle biopsies, murine skeletal and cardiac muscle were fixed in 2% paraformaldehyde (pH 6.8) for 1–2 minutes, followed by overnight incubation at 4°C with the primary antibodies (a 1:30 dilution was used for ab 2240, and 1:200 dilution for the abs RP291 and RP194, in blocking solution made of 3% bovine serum albumin and 0.05% Tween-20 in phosphate-buffered saline). Incubation with a mouse anti-rabbit biotin-conjugated secondary ab (1:120; Jackson Immunoresearch Laboratories, West Grove, PA) was followed by Cy-3-conjugated streptavidin (1:1000; Jackson Immunoresearch Laboratories). Controls consisted of sections treated in the absence of primary antibody. Neuromuscular junctions were revealed with Alexa-488-conjugated-α-bungarotoxin [α BTX] (1:40) (Molecular Probes, Eugene, OR). Slides were viewed on a Leica microscope-based imaging system using OpenLab imaging software (Quorum Technologies, St Catharines, ON).

### Western blot analysis and immunoprecipitation

Cardiac muscle tissue was homogenized in extraction buffer [1% Triton X-100; 0.1 mM EDTA; 0.1 mM EGTA; 50 mM Tris-HCl; pH 8.0; with protease inhibitors (Roche)] at 4°C. After a 30 second sonication, samples were centrifuged at 3000 × g for 30 seconds at 4°C. Protein samples (10 μg) were anayzed by sodium dodecyl sulfate-polyacrylamide gel electrophoresis (SDS-PAGE) using 10% (w/v) polyacrylamide gels, followed by electrotransfer to nitrocellulose. The blots were blocked in 10% BLOTTO (skim milk powder) in Tris buffered saline – Tween 20 (TBS-T) for 45 minutes at room temperature. Anti-CAR antibody was added in 10% BLOTTO at a dilution of 1:2500. Following incubation with peroxidase-labeled goat-anti-rabbit secondary antibody (Jackson Immunoresearch Laboratories), the signal was visualized by enhanced chemiluminescence (Amersham Pharmacia Biotech, Baie d'Urfe, QC).

Immunoprecipitation was carried out on cardiac muscle homogenates that had been pre-cleared with Protein-A Agarose slurry (Sigma), followed by overnight incubation at 4°C with RP291 or RP194. The samples were further incubated with Protein-A Agarose for 2 hours, washed twice with extraction buffer and then eluted with 2 X Laemmli SDS sample buffer with 5% mercaptoethanol. Following SDS-PAGE and electrotransfer, nitrocellulose membranes were probed with a primary polyclonal chicken anti-CAR (ChCT) in blocking solution (diluted 1:500) overnight at 4°C. Signal was revealed following incubation with a peroxidase-conjugated donkey anti-chicken IgY (Jackson Immunoresearch Laboratories) at a dilution of 1:2500 for 40 minutes, and enhanced chemiluminescence.

## Authors' contributions

CAS performed the immunoprecipitation, the immunoblotting, participated in the immunofluorescent staining experiments and drafted the manuscript. PCH and JN conceived the study, participated in its design and interpretation as well as drafting the manuscript. CA participated in the immunofluorescent staining experiments. KS prepared and characterized the isoform-specific antibodies. MS and GK participated in the design and interpretation of the studies. All authors read and approved the final manuscript.

## References

[B1] Bergelson JM, Cunningham JA, Droguett G, Kurt-Jones EA, Krithivas A, Hong JS, Horwitz MS, Crowell RL, Finberg RW (1997). Isolation of a common receptor for Coxsackie B viruses and adenoviruses 2 and 5. Science.

[B2] Tomko RP, Xu R, Philipson L (1997). HCAR and MCAR: the human and mouse cellular receptors for subgroup C adenoviruses and group B coxsackieviruses. Proc Natl Acad Sci U S A.

[B3] Roelvink PW, Lizonova A, Lee JG, Li Y, Bergelson JM, Finberg RW, Brough DE, Kovesdi I, Wickham TJ (1998). The coxsackievirus-adenovirus receptor protein can function as a cellular attachment protein for adenovirus serotypes from subgroups A, C, D, E, and F. J Virol.

[B4] Martino TA, Petric M, Weingartl H, Bergelson JM, Opavsky MA, Richardson CD, Modlin JF, Finberg RW, Kain KC, Willis N, Gauntt CJ, Liu PP (2000). The coxsackie-adenovirus receptor (CAR) is used by reference strains and clinical isolates representing all six serotypes of coxsackievirus group B and by swine vesicular disease virus. Virology.

[B5] Bergelson JM, Krithivas A, Celi L, Droguett G, Horwitz MS, Wickham T, Crowell RL, Finberg RW (1998). The murine CAR homolog is a receptor for coxsackie B viruses and adenoviruses. J Virol.

[B6] Honda T, Saitoh H, Masuko M, Katagiri-Abe T, Tominaga K, Kozakai I, Kobayashi K, Kumanishi T, Watanabe YG, Odani S, Kuwano R (2000). The coxsackievirus-adenovirus receptor protein as a cell adhesion molecule in the developing mouse brain. Brain Res Mol Brain Res.

[B7] Bruning A, Runnebaum IB (2003). CAR is a cell-cell adhesion protein in human cancer cells and is expressionally modulated by dexamethasone, TNFalpha, and TGFbeta. Gene Ther.

[B8] Cohen CJ, Shieh JT, Pickles RJ, Okegawa T, Hsieh JT, Bergelson JM (2001). The coxsackievirus and adenovirus receptor is a transmembrane component of the tight junction. Proc Natl Acad Sci U S A.

[B9] Hotta Y, Honda T, Naito M, Kuwano R (2003). Developmental distribution of coxsackie virus and adenovirus receptor localized in the nervous system. Brain Res Dev Brain Res.

[B10] Tomko RP, Johansson CB, Totrov M, Abagyan R, Frisen J, Philipson L (2000). Expression of the adenovirus receptor and its interaction with the fiber knob. Exp Cell Res.

[B11] Fechner H, Noutsias M, Tschoepe C, Hinze K, Wang X, Escher F, Pauschinger M, Dekkers D, Vetter R, Paul M, Lamers J, Schultheiss HP, Poller W (2003). Induction of coxsackievirus-adenovirus-receptor expression during myocardial tissue formation and remodeling: identification of a cell-to-cell contact-dependent regulatory mechanism. Circulation.

[B12] Kashimura T, Kodama M, Hotta Y, Hosoya J, Yoshida K, Ozawa T, Watanabe R, Okura Y, Kato K, Hanawa H, Kuwano R, Aizawa Y (2004). Spatiotemporal changes of coxsackievirus and adenovirus receptor in rat hearts during postnatal development and in cultured cardiomyocytes of neonatal rat. Virchows Arch.

[B13] Tallone T, Malin S, Samuelsson A, Wilbertz J, Miyahara M, Okamoto K, Poellinger L, Philipson L, Pettersson S (2001). A mouse model for adenovirus gene delivery. Proc Natl Acad Sci U S A.

[B14] Wan YY, Leon RP, Marks R, Cham CM, Schaack J, Gajewski TF, DeGregori J (2000). Transgenic expression of the coxsackie/adenovirus receptor enables adenoviral-mediated gene delivery in naive T cells. Proc Natl Acad Sci U S A.

[B15] Nalbantoglu J, Larochelle N, Wolf E, Karpati G, Lochmuller H, Holland PC (2001). Muscle-specific overexpression of the adenovirus primary receptor CAR overcomes low efficiency of gene transfer to mature skeletal muscle. J Virol.

[B16] Hurez V, Dzialo-Hatton R, Oliver J, Matthews RJ, Weaver CT (2002). Efficient adenovirus-mediated gene transfer into primary T cells and thymocytes in a new coxsackie/adenovirus receptor transgenic model. BMC Immunol.

[B17] Schmidt MR, Piekos B, Cabatingan MS, Woodland RT (2000). Expression of a human coxsackie/adenovirus receptor transgene permits adenovirus infection of primary lymphocytes. J Immunol.

[B18] Bergelson JM, Mohanty JG, Crowell RL, St John NF, Lublin DM, Finberg RW (1995). Coxsackievirus B3 adapted to growth in RD cells binds to decay-accelerating factor (CD55). J Virol.

[B19] Shafren DR, Bates RC, Agrez MV, Herd RL, Burns GF, Barry RD (1995). Coxsackieviruses B1, B3, and B5 use decay accelerating factor as a receptor for cell attachment. J Virol.

[B20] Shafren DR, Williams DT, Barry RD (1997). A decay-accelerating factor-binding strain of coxsackievirus B3 requires the coxsackievirus-adenovirus receptor protein to mediate lytic infection of rhabdomyosarcoma cells. J Virol.

[B21] Nalbantoglu J, Pari G, Karpati G, Holland PC (1999). Expression of the primary coxsackie and adenovirus receptor is downregulated during skeletal muscle maturation and limits the efficacy of adenovirus-mediated gene delivery to muscle cells. Hum Gene Ther.

[B22] Navenot JM, Villanova M, Lucas-Heron B, Malandrini A, Blanchard D, Louboutin JP (1997). Expression of CD59, a regulator of the membrane attack complex of complement, on human skeletal muscle fibers. Muscle Nerve.

[B23] Bowles NE, Dubowitz V, Sewry CA, Archard LC (1987). Dermatomyositis, polymyositis, and Coxsackie-B-virus infection. Lancet.

[B24] Sollerbrant K, Raschperger E, Mirza M, Engstrom U, Philipson L, Ljungdahl PO, Pettersson RF (2003). The Coxsackievirus and adenovirus receptor (CAR) forms a complex with the PDZ domain-containing protein ligand-of-numb protein-X (LNX). J Biol Chem.

[B25] Sanes JR, Schachner M, Covault J (1986). Expression of several adhesive macromolecules (N-CAM, L1, J1, NILE, uvomorulin, laminin, fibronectin, and a heparan sulfate proteoglycan) in embryonic, adult, and denervated adult skeletal muscle. J Cell Biol.

[B26] Dubois C, Figarella-Branger D, Pastoret C, Rampini C, Karpati G, Rougon G (1994). Expression of NCAM and its polysialylated isoforms during mdx mouse muscle regeneration and in vitro myogenesis. Neuromuscul Disord.

[B27] Perriard JC, Hirschy A, Ehler E (2003). Dilated cardiomyopathy: a disease of the intercalated disc?. Trends Cardiovasc Med.

[B28] Ito M, Kodama M, Masuko M, Yamaura M, Fuse K, Uesugi Y, Hirono S, Okura Y, Kato K, Hotta Y, Honda T, Kuwano R, Aizawa Y (2000). Expression of coxsackievirus and adenovirus receptor in hearts of rats with experimental autoimmune myocarditis. Circ Res.

[B29] Sasse A, Wallich M, Ding Z, Goedecke A, Schrader J (2003). Coxsackie-and-adenovirus receptor mRNA expression in human heart failure. J Gene Med.

[B30] Fechner H, Haack A, Wang H, Wang X, Eizema K, Pauschinger M, Schoemaker R, Veghel R, Houtsmuller A, Schultheiss HP, Lamers J, Poller W (1999). Expression of coxsackie adenovirus receptor and alphav-integrin does not correlate with adenovector targeting in vivo indicating anatomical vector barriers. Gene Ther.

